# Mr. Balfour on Medicine

**Published:** 1895-05-25

**Authors:** 


### The Hospital

An Institutional Journal of

The Medical Sciences and Hospital Administration, with

AN EXTRA NURSING SUPPLEMENT.

Vol. XVIII.—No. 452.] [May 25, 1895.

### Mr. Balfour on Medicine.

It is, perhaps, a little surprising, though quite undeniable, that Mr. A. J. Balfour, the leader of Her Majesty's Opposition, has taken a broader, more philosophical, juster, and more practical view of the quality and service of modern medicine than many modern medical men themselves. When we are told, as we often are, to endeavour to "see ourselves as others see us," it is usually assumed that the sight will be mortifying to our self-satisfaction, and conducive to a growth in much-needed humility. But when we borrow Mr. Balfour's eyes for the purposes of self-inspection, we find ourselves, not mortified, but genially and delightfully expanding in all directions, like spring flowers in unexpected sunshine. That Mr. Balfour's is the strictly accurate measure of our merit we need not claim. The generosity of his heart has lent wings to his appreciation. Nevertheless, we feel the summer sunshine of the statesman's praise exceedingly welcome, and we are sure that its effect upon all sorts and conditions of men in our sternly-toiling profession will be entirely good.

It is strange, more than strange, how slowly the assured medical and physiological sciences which constitute the elements of the best modern practice have filtered down to the mind of even the educated layman. The uncultured know nothing at all of our science and its objects and aims. Nay more, many, even among medical men, still practice by "rule of thumb," and their "rule of thumb" is the surgery and dispensary experience of half a century ago. Of scientific facts they may know a little, because they cannot help it. But of the large general principles of science they know nothing at all; whilst of the inquiring, investigating, applying temper of science they know, if that were possible, not nothing, but less than nothing. It is here that Mr. Balfour, and before him Mr. Gladstone, Lord Salisbury, and other statesmen, have put us to the blush. Mr. Balfour could not, probably, detect a cardiac murmur with the stethoscope or even open an abscess with a curved bistoury. And because the latest fledged of our medical neophytes can do these elementary things better than the statesman, they read his philosophic words with a pitiful sense of patronising superiority. Instead of that, they should hear in such words trumpet-tongued incentives to thought, to enlargement of mind, to high and wide purpose, to strenuous achievement on behalf of the life, development, happiness, and increasing efficiency and prosperity of the whole race of man.

It is to be confessed with sadness that the average medical man is not enthusiastic. The world is enthusiastic about medical progress; but many medical men kill their own and their brethren's enthusiasm with the frost of a narrow and incompetent criticism. Not a few members of our profession think they are critical because they know. The truth is they are critical because they do not know, because knowing a little they do not know enough, and because not knowing enough they do not thoroughly understand what they do know. Not twenty years ago it was considered the surest of all signs of scientific superiority in medicine to be an agnostic or a sceptic in treatment. No words can adequately express the just contempt of the healthy mind for imbecility of this order. Mr. Balfour's enthusiasm on our behalf; his appreciation of the science to which we have already attained and of the practical skill based upon it; his ardour of vitality and progress when he looks forward to the still greater heights of knowledge and practical skill which are waiting to be achieved, may well rouse us from the ignoble inefficiency of mere futile criticism to inspired and unconquerable determination to carry the banner of high science and practice into every department of the life and activity of the world.

The rank and file of medicine are too much neglected by those in position and authority. It needs a man of large, generous, and statesmanlike temper to recognise what is being done by the "great unknown" among us. "In every district, in every parish, in almost every street," Mr. Balfour tells us, "medical men lavish the treasures of their time and skill on those who, from their worldly circumstances, are very ill able to repay them." "The treasures of their skill." It is but the bare truth. Notwithstanding that many men outside the boundaries of professional criticism still practise the "rule of thumb" methods of a past generation, it is nevertheless true that there are thousands of medical men, fast rising to middle life, who have attained to remarkable heights of science and skill, who "lavish the treasures" of their skill upon those who can make them little or no return, and yet who are looked upon by those more fortunately placed in medicine with the eye of a cynical and unrecognising disdain.

Most injurious is all this to the growth and development of general medical science and skill. We cannot but thank Mr. Balfour for his high appreciation of the work of our profession as a whole; and whilst doing so, we venture to ask those of our brethren who are of mature professional age, and who have attained to positions of influence and authority, to borrow from the statesman some of his generous enthusiasm. Armed with that they will not fail to rouse and inspire all ranks among us to aim steadily at the highest, and to feel assured that in so doing whoever else may see weakness and failure in the efforts of the obscure, their own brethren will discern and judge, and give "honour to whom honour is due."

